# Recent Advances in Elucidating the Genetic Mechanisms of Nephrogenesis Using Zebrafish

**DOI:** 10.3390/cells4020218

**Published:** 2015-05-27

**Authors:** Christina N. Cheng, Valerie A. Verdun, Rebecca A. Wingert

**Affiliations:** Department of Biological Sciences, Center for Zebrafish Research, and Center for Stem Cells and Regenerative Medicine, University of Notre Dame, Notre Dame, IN 46556, USA; E-Mails: ccheng2@nd.edu (C.N.C.); verdunv12@alumni.nd.edu (V.A.V.)

**Keywords:** *aPKC*, kidney development, *hnf1ba/b*, *mecom*, nephron segmentation, renal progenitor, *sim1a*, tubulogenesis, vertebrate, zebrafish

## Abstract

The kidney is comprised of working units known as nephrons, which are epithelial tubules that contain a series of specialized cell types organized into a precise pattern of functionally distinct segment domains. There is a limited understanding of the genetic mechanisms that establish these discrete nephron cell types during renal development. The zebrafish embryonic kidney serves as a simplified yet conserved vertebrate model to delineate how nephron segments are patterned from renal progenitors. Here, we provide a concise review of recent advances in this emerging field, and discuss how continued research using zebrafish genetics can be applied to gain insightsabout nephrogenesis.

## 1. Introduction

The vertebrate kidney is an essential organ that functions to remove metabolic waste, produce hormones, and maintain homeostasis (e.g., fluid, pH, blood pressure) within the body. These important physiological roles are primarily achieved by the nephrons, which are the fundamental structural and functional units of the kidney [1,2]. Each nephron is composed of a glomerular blood filter attached to a segmented epithelial tubule that facilitates the modification of filtrates and production of urine [1,2]. This waste is then channeled from the nephrons through a highly branched collecting duct system to the urinary bladder for excretion [1,2].

Renal ontogeny in higher vertebrates is typically characterized by the formation and subsequent degradation of up to three types of kidneys, all comprised of nephrons, which arise over time from the intermediate mesoderm (IM) [3]. The first kidney-like structure generated in an embryo is the pronephros [3]. Upon the degradation of the pronephros, the mesonephros functions transiently until the formation of the metanephros, which serves as the adult kidney [3]. Organogenesis of these progressive kidney forms requires the precise reciprocal interactions of renal progenitor populations and their integration with other tissues, such as the developing vasculature (reviewed in [4,5]). Many studies utilizing animal and cell culture models have provided further insight into the phenomenon of kidney development, such as the identity of crucial regulatory genes [6] and their transcriptional networks [7]. The molecular features that define the renal progenitors have been increasingly well characterized over the last several years (reviewed in [8,9,10,11,12]). Further, there has been steady progress in delineating the developmental dynamics of renal progenitor expansion, self-renewal, and the mechanisms that underlie differentiation of nephron precursors [13,14,15].

Despite these advances, certain aspects of nephrogenesis, including the formation of a lumen (reviewed in [16,17]) and segmentation patterning (reviewed in [18,19,20]), remain poorly understood. In part, as the mammalian kidney can contain thousands to millions of nephrons, the structural complexity of this vital organ has ultimately complicated the interrogation of the genetic and molecular mechanisms that dictate renal ontogeny among higher vertebrates [20]. The importance of understanding nephrogenesis, however, is central to progress in addressing renal abnormalities and their origins during development [21]. The wide spectrum of congenital anomalies of kidney and urinary tract (CAKUT) represent approximately 30% of malformations diagnosed in the prenatal period, and are a major cause of morbidity in children [22,23]. Continued identification of the genes and morphogenetic processes that control kidney formation is needed to appreciate the basis of these diverse congenital defects and formulate improved diagnosis and interventions for such conditions.

## 2. Modeling Vertebrate Nephrogenesis Using the Zebrafish Pronephros

In recent years, emerging research has provided significant evidence that the genetic composition of the nephron is conserved between kidney forms in mammals, avians, reptiles, amphibians, and fish [24,25]. Lower vertebrates, such as fish and amphibians, exhibit overt differences in renal development compared to mammals: namely, they form a functional pronephros, and they never develop a metanephros, instead utilizing the mesonephros as their final kidney structure [26]. However, through gene expression profiling that was largely based on solute transporter protein encoding factors, a number of studies have determined that the nephron segmentation pattern within the assorted pronephric, mesonephric, and metanephric kidney forms is fundamentally similar between these organisms [24,25]. The number of nephrons and their anatomical arrangements differ substantially, though, with the simplest organizations present in pronephric kidneys and the most complex in metanephric kidneys [24,25,26]. In light of the conserved nature of the vertebrate nephron, the pronephros is an appealing setting to study the fundamental genetic programs that direct nephrogenesis, as it has both a low number and simple arrangement of nephrons [27].

**Figure 1:**
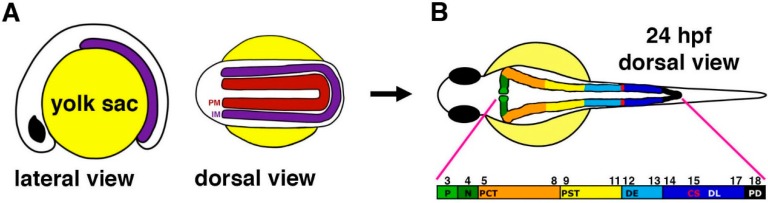
Anatomy of the zebrafish pronephros. (**A**) The zebrafish pronephros develops from bilateral fields of renal progenitors (purple) that emerge from the intermediate mesoderm (IM) and are located lateral to the paraxial mesoderm (PM, red) that gives rise to the somites (denoted by their number along the embryo axis respective to each nephron segment in panel B); (**B**) At 24 hours post fertilization (hpf), the two nephrons have formed and consist of a series of segments that include: podocytes (P, light green) that contribute to the blood filter, neck (N, dark green), proximal convoluted tubule (PCT, orange), proximal straight tubule (PST, yellow), distal early (DE, sky blue), corpuscles of Stannius (CS, red), distal late (DL, dark blue), and pronephric duct (PD, black). The numbers indicate the position of each nephron segment with respect to the somites. [Figure adapted from [45], through terms of the Creative Commons License of the Authors].

Among vertebrate model organisms, the pronephros of the zebrafish, *Danio rerio*, is particularly advantageous due to the embryonic transparency and rapid external development [28], as well as the wealth and versatility of genetic, cellular, and physiological manipulations that can be performed [29,30,31,32,33,34,35,36,37,38,39]. Specifically, the zebrafish pronephros consists of only two linear nephrons that are adjoined proximally by a blood filtering glomerulus and distally by the cloaca [40] (Figure 1). The glomerulus is structurally analogous to the typical filter apparatus in vertebrates, being comprised of capillaries that are surrounded by podocytes, which are specialized renal epithelial cells that help to comprise a sieve through which filtrate is collected [40,41,42,43]. Moreover, a series of proximal and distal tubule segments are present within the zebrafish pronephric nephrons [41,42], thus providing a simplified yet genetically tractable model system for investigating a wide array of renal developmental processes (recently reviewed in [43,44]) (Figure 1).

Research has previously shown that the functions of many genes known to be important during mammalian renal ontogeny are conserved in the zebrafish [43,44]. Consequently, the zebrafish is quite amenable for the *in vivo* elucidation of the pathways that regulate kidney development, and has been a useful context to study the molecular regulation and mechanical aspects of glomerular assembly, for example [46,47,48,49,50,51]. In the following sections, this review will highlight and explore the most recent discoveries made regarding tubulogenesis and segmentation events within the zebrafish pronephros.

## 3. Tubulogenesis of the Zebrafish Pronephros

Across species, renal progenitors are mesenchymal in nature, and ultimately need to undergo a mesenchymal to epithelial transition (MET) in order to form nephron tubules. Interestingly, there is only a rudimentary understanding of various tubulogenesis events, such as the timing of lumen formation, polarity establishment, as well as growth and morphogenesis of the nascent nephron. In general, the acquisition of apical-basal polarity by precursor cells is considered a defining moment during the establishment of tubular organs across vertebrates [52]. Throughout this phenomenon, distinct apical and basolateral domains are created within the cells. One example of a polarization component is the conserved ternary polarity complex, consisting of atypical protein kinase C (aPKC), Par-3, and Par-6, which localizes to the apical cell membrane [53]. Upon polarization, the apical surface of the cell is located next to the lumen, while the extracellular matrix (ECM) is adjacent to the basal cell membrane [54,55].

In zebrafish, the pronephric nephrons arise from bilateral renal progenitor fields that emerge from the IM [40,48] (Figure 1). The renal progenitors exhibit dynamic spatiotemporal gene expression patterns during early zebrafish embryogenesis, prior to the establishment of the distinctive segmentation pattern within the nephrons at 24 hours post fertilization (hpf) [41,42]. Further, tubulogenesis from renal progenitors in the IM occurs rapidly, being completed by approximately 24 hpf [43]. While studies have sought to elucidate the molecular pathways that underlie tubulogenesis in many zebrafish tissues, the precise timing and mechanisms of lumen formation and polarity establishment in the pronephros were not scrutinized. Previous research has noted that the loss of proteins associated with the aPKC complex can negatively alter the establishment of the lumen in various tissues, such as the gut [53]. Moreover, expression of the iota (ι) and zeta (ξ) aPKC protein isoforms have been documented throughout murine renal organogenesis [56], though the function of these proteins have not yet been identified with regards to nephron tubulogenesis until now [57].

**Figure 2:**
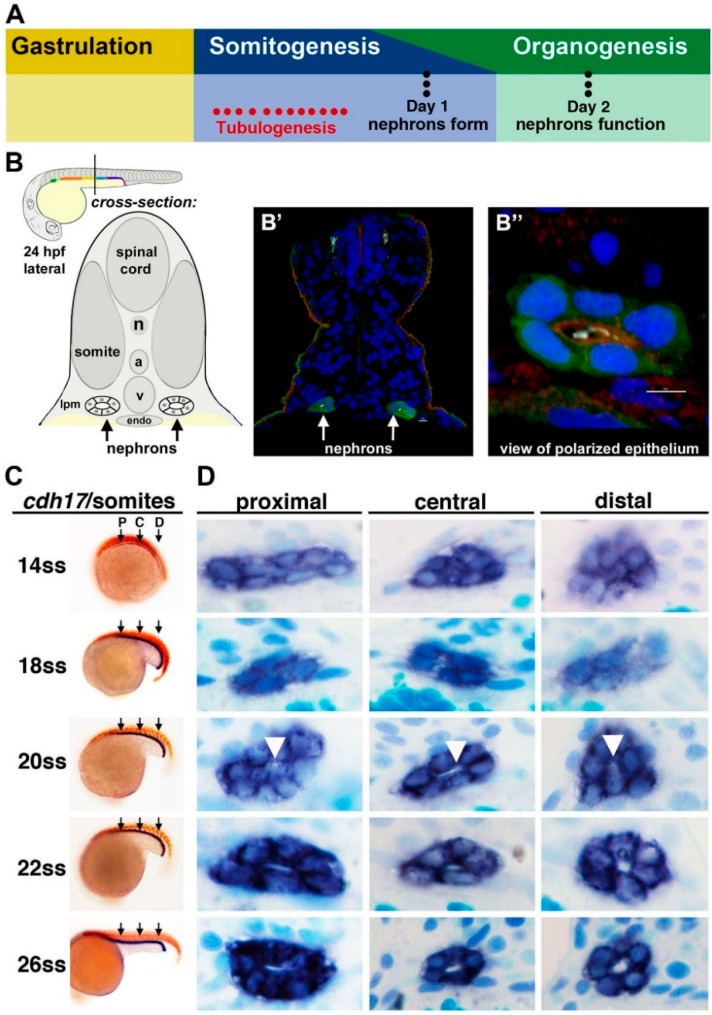
Tubulogenesis of the zebrafish pronephros. (**A**) The timing of tubulogenesis is coincident with the stages of somitogenesis and organogenesis of the embryo; (**B**–**B**”) At 24 hours post fertilization (hpf), the two nephrons have formed distinct tubule lumens that can be detected by immunofluorescence to detect green fluorescent protein (GFP), acetylated tubulin (light blue), Prkc ι/ξ (red) and nuclei (DAPI, blue); (**C**, **D**) The precise onset of tubulogenesis occurs at the 20 ss, indicated by white arrowheads, along the proximal, central, and distal regions of the nephron territory, with progressive enlargement of the luminal space at 22 and 26 ss. Abbreviations: aorta (a), lateral plate mesoderm (lpm) notochord (n), somite stage (ss) cardinal vein (v), [Figure adapted from [43,57], through terms of the Creative Commons License of the Authors].

Recently, the timing of nephron tubulogenesis and polarity establishment in the zebrafish pronephros were ascertained through histological and immunofluorescence studies [57]. The authors demonstrated that renal progenitors undergo a MET to form a lumen at approximately the 20 somite stage (ss) [57] (Figure 2), which coincides with the regional expression of various tight junction components in the pronephros, such as *cldn15a* and *cldn8* [58].

**Figure 3:**
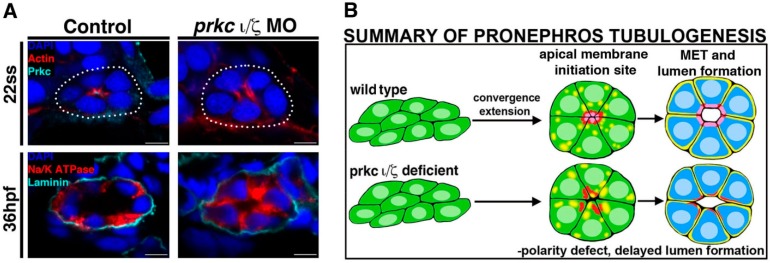
Deficiency of Prkcι/ξ disrupts nephron epithelial polarity. (**A**) At the 22 ss, actin is mislocalized in Prkcι/ξ deficient embryos, though lumen formation eventually occurs by 36 hpf, accompanied by normal laminin distribution at the basal lamina though Na^+^/K^+^ ATPase distribution remains mislocalized at this time point. (**B**) Summary of phenotypes in Prkcι/ξ deficient embryos compared to wild-type embryos. Abbreviations: hours post fertilization (hpf), mesenchymal to epithelial transition (MET), morpholino (MO) somite stage (ss). [Figure adapted from Reference [57], through terms of the Creative Commons License of the Authors].

Additionally, it was determined that changes in protein localization lead to the eventual distinction between the apical (Prkcι/ξ^+^) and basolateral (Na^+^/K^+^ ATPase^+^) regions [57]. Furthermore, the functional significance of these Prkc isoforms *in vivo* was discovered through single and double morpholino knockdowns in wild-type embryos [57]. In contrast to single Prkc knockdown embryos, double Prkcι/ξ morphants had abnormal localization of actin (Figure 3) and Na^+^/K^+^ ATPase, and the protein Ezrin, Radixin, and Moesin (p-ERM) and Prkc proteins were absent from the pronephros, suggesting redundant roles of Prkcι/ξ during nephron tubule polarization [57]. Since previous research has also indicated that renal diseases (e.g., polycystic kidney disease, PKD) are associated with epithelial polarity defects [59], it would be interesting in future studies to further interrogate how renal progenitors are affected by disruptions in Na^+^/K^+^ ATPase and p-ERM localization. In addition, investigating whether Prkcι/ξ deficiency, or the combined deficiency of Prkcι/ξ and other polarity regulators, develop cysts or show altered epithelial tubule regeneration could provide useful models to study certain aspects of kidney disease [57,58,59].

Interestingly, while further experiments revealed that apicobasal polarity was not present in doubly deficient Prkcι/ξ embryos, a lumen eventually formed by 36 hpf and nephron segmentation was normal [57] (Figure 3). Together, these data indicated that the attainment of cell polarity is indeed dependent on Prkcι/ξ function, but nephron lumen formation and segment specification are not [57]. In terms of the delayed lumen development, it is possible that other polarity complexes and junction proteins provide redundant or partially redundant roles that are still adequate for this event to occur in the absence of Prkcι/ξ, therefore providing an interesting basis for future tubulogenesis studies.

Pronephric morphogenesis events were also evaluated in Prkcι/ξ morphants [57]. Typically, between 48 and 72 hpf, the proximal convoluted tubule (PCT) will undergo convolutions in a wild-type setting. However, the PCT remained anatomically linear following the loss of Prkcι/ξ, and findings from dextran uptake assays indicated that the PCT cells were unable to uptake and clear the dextran from the body, signifying the potential absence of fluid flow and proper kidney function in these morphants [57].

Notably, the abrogation of Prkcι/ξ not only disrupted tubulogenesis resulting in decreased kidney function, but the role of these aPKC isoforms in the maintenance of epithelial identity was revealed as well [57]. For example, prior research showed that the expression of *wt1a*, a glomerular podocyte gene, is restricted from the neck and proximal tubule regions of the nephron by *pax2a* [47]. Significantly, after the loss of Prkcι/ξ, *pax2a* and podocyte genes (*wt1a*, *wt1b*, *podxl*) were misexpressed throughout the pronephros [57]. However, the additional knockdown of *pax2a,* but not *wt1a,* in combination with that of Prkcι/ξ, was able to rescue the ectopic expression of the podocyte specific genes [57]. These results ultimately implied that Prkcι/ξ inhibits *pax2a* in order to preserve normal gene expression throughout the pronephros, thus maintaining epithelial cell type identities [57].

Overall, this study provides new insights into the acquisition of polarity among renal progenitors during MET in terms of the Prkcι/ξ isoforms, which exhibit partially redundant functions. Moreover, the loss of polarity due to the absence of Prkcι/ξ caused ectopic expression of important renal transcription factors, including *pax2a* and *wt1a*, within the pronephros. These findings are indicative of either (1) an incomplete transition of precursor cells from a mesenchymal to an epithelial state or (2) the re-expression of renal development genes in specified cells. Additionally, a novel pathway involving the repression of *pax2a* by the Prkcι/ξ proteins is thought to regulate gene expression, thereby maintaining pronephric epithelial cell identities. However, it is currently unknown whether these effects are direct or indirect, leaving intriguing avenues for future investigations.

## 4. Broad Segmental Patterning of the Nephron Tubule is Reliant on *Hnf1ba/b*

Previous studies have shown that orthologs of many key genes, implicated to be important during murine renal organogenesis, are similarly expressed in the zebrafish kidney. As such, numerous transcription factors have been mapped to the emerging renal progenitor domains [41,42], though the functional roles of most remain an enigma [43,44]. Accordingly, how renal progenitor lineages arise and then become specified into the characteristic cell types of each nephron segment is an ongoing topic of research in the nephrology field [18,19,20]. As previously mentioned, research has noted that genetic conservation exists between the segmentation patterning of the mammalian, *Xenopus*, and zebrafish nephrons, namely the proximal convoluted and straight tubules (PCT, PST) as well as the distal early and late (DE, DL) segments [24,60]. The anatomical simplicity of the zebrafish pronephros has enabled the genetic analysis of how segmentation patterning occurs *in vivo* as revealed by several recent studies on a series of transcription factors expressed during nephrogenesis.

Recent work has shown that the homeodomain transcription factors encoded by the genes *hepatocyte nuclear factor-1 beta a/b (hnf1ba/b)* are required broadly for segmentation across the nephron [61]. Through a series of gene knockdown studies, embryos deficient in *hnf1ba/b* were shown to form pronephric tubules, but these lacked expression of all segment markers [61]. Acridine orange staining was performed to assess if apoptosis occurred but significant cell death was not identified, thereby suggesting a defect in segment patterning [61]. Nephron sectioning revealed that epithelial cells were present in the tubule, suggesting that the IM cell transition into an epithelial state is not affected in *hnf1ba/b*-deficient embryos [61]. Expression of the early acting renal progenitors *pax2a*, *pax8*, *lhx1a*, and *mecom* were examined in *hnf1ba/b*-deficient embryos [61]. In wild-type embryos, these transcripts are lost in the proximal pronephros after the 15 ss; however, in *hnf1ba/b*-deficient embryos, they were not downregulated and instead remained in proximal regions at 24 hpf [61]. This data suggests that Hnf1b transcription factors act to define the spatiotemporal localization of *pax2a*, *pax8*, *lhx1a*, and *mecom* prior to the emergence of mature nephron segmentation at 24 hpf [61].

Interestingly, ectopic podocyte formation was observed in *hnf1ba/b*-deficient embryos [61]. Podocyte progenitor cells form in zebrafish around the 12 ss and express the *wt1a* and *wt1b* transcription factors [49,50]. The podocyte progenitor cells migrate toward the midline and also display expression of differentiation markers such as *nephrin* and *podocin* [50]. *hnf1ba/b*-deficient embryos at 24 hpf displayed an expanded podocyte domain in the region that the PCT segment normally occupies [61]. Similarly, at 48 hpf, *nephrin* and *podocin* positive cells were found in *hnf1ba/b*-deficient embryos; however, normal midline fusion did not occur [61]. To investigate the timing of this fate change, *hnf1ba/b*-deficient embryos were examined at the 15 ss and showed an increased number of *wt1b^+^* cells, indicating increased podocyte progenitor formation [61]. Double *in situ* hybridization revealed that the *wt1b* and *hnf1ba* domains do not overlap in wild-type embryos at the 15 stages, and thus, mark distinct regions of the nephron [61]. Taken together, these results suggest that Hnf1b factors act in proximal cells to inhibit podocyte formation. To determine if ectopic podocyte formation in *hnf1ba/b*-deficient embryos was dependent on retinoic acid (RA), RA signaling was blocked using 4-diethylaminobenzaldehyde (DEAB) in *hnf1ba/b*-deficient embryos from the early gastrula to the 15 ss. Data from a previous study observed a loss of podocytes and tubules comprised of only distal segments in wild-type embryos treated with DEAB [41]. In *hnf1ba/b*-deficient embryos at 24 hpf, *wt1b^+^* podocyte cells were absent when treated with DEAB, suggesting that Hnf1B factors act downstream of RA signaling, since the deficiency of these factors was unable to rescue podocyte loss in the context of the DEAB-mediated abrogation RA synthesis [61]. Normally, DEAB-treated wild-type embryos display an expansion of DL tubule marker *slc12a3*; however, this effect was prevented in *hnf1ba/b*-deficient embryos [61]. 

Additionally, morpholino knockdown of *wt1a* and *rbpj* in *hnf1ba/b*-deficient embryos was performed to assess the epistatic relationship between *hnf1ba/b*, *wt1a,* and *rbpj* [61]. It was previously demonstrated that the formation of podocyte progenitors is dependent on *wt1a* and *rbpj*, the latter which encodes a transcriptional mediator of the Notch pathway [50]. Embryos deficient in *hnf1ba/b*, *wt1a,* and *rbpj* failed to express *wt1b*; however, *hnf1ba/b*-deficient embryos that did not undergo morpholino knockdown of *wt1a* and *rbpj* did express *wt1b* [61]. These results indicate that the development of ectopic podocytes in *hnf1ba/b-*deficient animals is dependent on *wt1a* and Notch signaling [61]. *jagged2a*, which encodes a Notch ligand involved in podocyte formation, also failed to downregulate in the proximal pronephros of *hnf1ba/b-*deficient embryos [61]. In addition, overexpression of *hnf1ba* mRNA did not suppress *wt1a^+^* and *wt1b^+^* cell formation [61]. Taken together, the authors hypothesized that Hnf1b factors act upstream or in parallel to *wt1a* and Notch signaling during podocyte formation.

In evaluating possible downstream targets of *hnf1ba/b* signaling, the authors also explored the relationship of these factors with the *iroquois homeobox 3b (irx3b)* transcription factor, which has been previously shown to be essential for DE segment differentiation [42]. In gene expression studies to address the epistatic relationship between *hnf1ba/b* and *irx3b*, the researchers found that *irx3b* was not expressed in *hnf1ba/b*-deficient embryos at 24 hpf [61]. To determine if *irx3b* is required to maintain *hnf1ba* expression in the DE segment, wild-type embryos were injected with *irx3b* morpholinos and the expression of *hnf1ba* was examined at 24 hpf. *irx3b*-deficient embryos displayed downregulated expression of *hnf1ba* in the DE [61]. This data suggests that Hnf1b factors are initially required to induce *irx3b* expression, but then Irx3b is needed later to maintain *hnf1ba* expression in the DE [61].

Taken together, this valuable study demonstrated novel roles of the Hnf1b factors as major regulators of nephron segmentation and podocyte development. *HNF1b* mutations in humans are linked to cyst formation and are associated with severe congenital abnormalities of the kidney, which suggests that *HNF1b* plays an early role in nephrogenesis. The insights gained about the Hnf1b factors from this study may ultimately help direct research about kidney cysts and renal birth defects in patients with Hnf1b deficiencies.

## 5. Segment-Specific Roles in Patterning of the Nephron Tubule: *mecom* and *sim1a*

Prior research has documented the expression of the *mecom* zinc finger transcription factor, which is the product of a splice variant from the *myelodysplastic syndrome 1* and *ecotropic virus integration site 1* genes, in the distal regions of the developing *Xenopus* and zebrafish kidneys [42,62,63]. Moreover, the conserved gene *single minded family bHLH transcription factor 1a* (*sim1a*) has also been noted in the murine kidney and zebrafish renal progenitors [48,64]. These findings, in addition to the known roles of *mecom* and *sim1a* in other vital developmental processes such as hematopoiesis and neurogenesis, respectively, make them intriguing gene candidates for the regulation of nephron segmentation [65,66,67]. Consequently, since the activities of these genes during nephrogenesis were unknown, recent studies have addressed these topics and revealed the functional significance of *mecom* and *sim1a* to date [45,68]. Interestingly, both *mecom* and *sim1a* expression was found to be dynamic during early zebrafish renal ontogeny, where mRNA transcripts of these genes were found to be exclusively in the caudal domain of *pax2a+* renal progenitors based on whole mount *in situ* hybridization (WISH) expression experiments [45,68]. However, by the 28 ss, *mecom* expression became localized to the DL segment and pronephric duct [68] while *sim1a* was first located solely in the proximal regions of the nephron at the 22 ss before becoming restricted to the teleost specific endocrine glands, known as the corpuscles of Stannius (CS), at the 28 ss [45]. Therefore, these results revealed the overall refinement and dynamism of gene expression patterns during nephrogenesis. 

**Figure 4:**
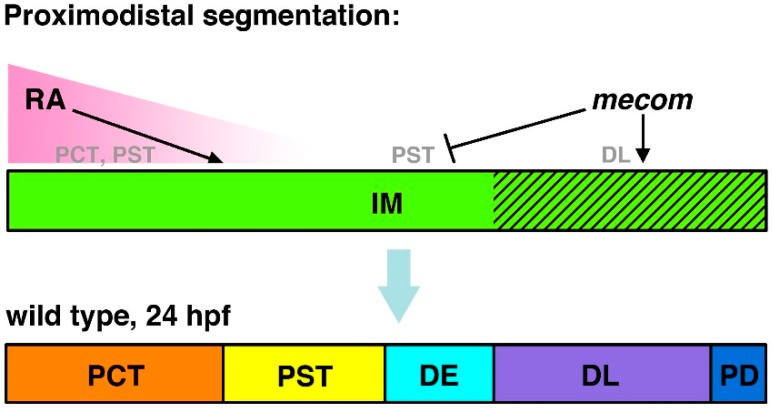
Model of *mecom* functions in nephron segmentation. *mecom* expression is proposed to promote DL formation and inhibit PST fates during proximodistal segment patterning. These activities act in opposition to RA signaling, which promotes PCT and PST formation and has been speculated to inhibit distal fates. Abbreviations: distal early (DE), distal late (DL), intermediate mesoderm (IM), proximal convoluted tubule (PCT), pronephric duct (PD), proximal straight tubule (PST), retinoic acid (RA). [Figure adapted from [68], through terms of the Creative Commons License of the Authors].

During loss of function experiments, these studies found that nephron segmentation defects were elicited following the knockdown of either *mecom* or *sim1a* [45,68]. Specifically, *mecom* morphants display expanded PST, which led to a corresponding distal shift of the DE, as well as a reduced DL segment [68]. Thus, it is likely that *mecom* regulates normal segmentation patterning by restricting PST fates and promoting DL cell types [68] (Figure 4).

**Figure 5:**
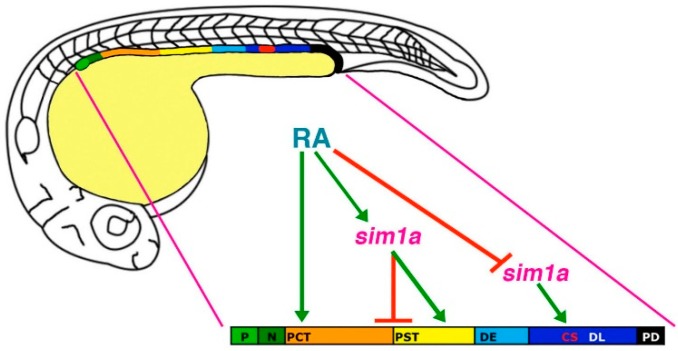
Model of *sim1a* functions in nephron segmentation. *sim1a* is necessary to promote PST and CS formation and inhibit PCT fates during segment patterning in the zebrafish pronephros. These activities occur downstream of RA signaling, which promotes PCT and PST formation and expression of *sim1a* in these respective renal progenitor domains. Further, RA signaling inhibits *sim1a* expression in CS progenitors, placing it as a negative regulator upstream of *sim1a* during establishment of the CS lineage. Abbreviations: corpuscle of Stannius (CS), distal early (DE), distal late (DL), neck (N), podocytes (P), proximal convoluted tubule (PCT), pronephric duct (PD), proximal straight tubule (PST), retinoic acid (RA). [Figure adapted from [45], through terms of the Creative Commons License of the Authors].

In comparison, the loss of *sim1a* caused different morphant phenotypes with regards to segmentation. In the absence of *sim1a,* the PCT became expanded while PST and CS populations were completely abrogated [45]. Despite these drastic alterations in the proximal nephron region, the distal segments remained unchanged [45]. Therefore, these data suggest that *sim1a* is required for PST and CS formation, and could be regulating the PCT/PST by inhibiting PCT cell fate or by stimulating a PST-specific gene program [45] (Figure 5). Furthermore, both *mecom* and *sim1a* morphants were characterized by the appearance of pericardial edema and decreased renal clearance functionality as assessed by dextran uptake assays [45,68]. Together, these findings revealed previously unknown roles for *mecom* and *sim1a* during the patterning of the nephron and kidney function.

These studies also sought to establish the relationship between *mecom* and *sim1a* to other pathways that are known to be crucial for renal ontogeny. Past research has identified retinoic acid (RA) as a major player in the regulation of nephrogenesis [41,42]. In general, RA target cells will express retinoic acid receptors (RXR, RAR) on their cellular membrane. Secreted RA molecules are then able to interact with RXR/RAR, leading to its internalization where it is able to regulate gene transcription by binding to retinoic acid response elements (RAREs) in the nucleus [69]. Relevant to renal development, a RA gradient is created by the secretion of this signaling molecule from the anterior paraxial mesoderm (PM) [24,69]. This important environmental cue, in turn, influences the renal progenitor field by inducing proximal identities while inhibiting distal fates during early somitogenesis [24,41,42]. Therefore, dose-dependent expansions of proximal segments and reductions of distal regions within the nephron are observed during exogenous RA treatment. In contrast, as previously mentioned, inhibiting RA synthesis with DEAB has opposite effects where the distal domains become expanded at the expense of the proximal segments [41,42]. In accordance with the literature, *mecom* expression in the distal nephron regions was reduced when wild-type embryos were treated with RA, while DEAB greatly expanded the *mecom* domain at the 28 ss, implying that RA is negatively regulating *mecom* during nephrogenesis [68] (Figure 4). Similarly, as expected, *sim1a* expression within the proximal domain became expanded following exogenous RA treatment, and was reduced by DEAB at the 18 ss [45]. Additionally, at the 28 ss when *sim1a* is only exhibited in the CS, exogenous RA distalized *sim1a* expression while DEAB expanded and proximalized the *sim1a* domain [45]. A RARE consensus sequence was also found in the putative promoter region of *sim1a*, suggestive of direct interactions with RA [45]. Notably, when *sim1a* morphants were treated with RA, which should promote proximal identity, the PST was still absent [45]. Moreover, the *sim1a* deficiency induced loss of the CS was unable to be rescued by DEAB [45]. Together, this data provided additional support that *sim1a* is indeed essential downstream of RA for the formation of the PST and CS [45] (Figure 5).

Significantly, these studies ultimately provided novel insights into the genetic control of nephron patterning. In particular, *mecom* was shown to play a fundamental role in proximodistal segmentation events, while *sim1a* activity is required for PST and CS formation. Evidence supporting the modulation of these gene transcripts by RA during kidney development was discovered as well, further revealing the intricacies of the regulatory network that governs this process. However, it is unclear whether such interactions are direct or indirect, thus requiring future studies that will focus on determining potential gene targets and binding partners, for instance. Also, given the effects of *mecom* and *sim1a* deficiency on the PST, it would be interesting to interrogate the potential relationship between these two genes in the regulation of this particular segment in nephrogenesis [45,68]. Nevertheless, these studies provide the basic foundation for future evaluations of *mecom* and *sim1a* functions in higher vertebrates, such as the mouse, during renal organogenesis.

## 6. Conclusions/Outlook

The zebrafish pronephros provides a simplified renal system for the identification and functional analysis of genes that are essential for nephrogenesis. In recent years, the advantages for relevant biomedical research using the zebrafish pronephros have been highlighted by the demonstration of its conserved nephron anatomy with other vertebrates [42,43,44]. Here, we have discussed how recent studies have provided new insights into the processes of apical-basal polarity establishment during MET in nephron progenitors [57,58], and identified roles for the transcription factors *hnf1ba/b* [61], *mecom* [68], and *sim1a* [45] in nephron segmentation. Looking forward, there is a wealth of genetic tools that can be employed to further delineate nephrogenic processes using zebrafish, including reverse genetics using genome editing and chemical genetics [29,37]. These methods provide excellent opportunities to rapidly assess the functional roles of genes expressed by renal progenitors and to identify currently unappreciated factors that impact nephron pattern formation. Additionally, the zebrafish pronephros can be used to study several aspects of nephron growth and morphogenesis, as the linear tubules undergo elongation and convolutions subsequent to the establishment of segment pattern [43]. In the coming years, understanding the mechanisms of renal regeneration [70] and how renal fates can be manipulated, such as through their *in vitro* production from pluripotent cell lines [71], will provide ways to model the cell biology of renal diseases and possibly create novel tools for regenerative medicine. Continued genetic research in animal models like the zebrafish will continue to support these endeavors by providing necessary clues to achieving these goals.
